# Sedum formosanum subsp. miyakojimense (Crassulaceae), a new subspecies from Miyako-jima Island of the Ryukyu Islands, Japan

**DOI:** 10.3897/phytokeys.148.48957

**Published:** 2020-05-26

**Authors:** Takuro Ito, Chih-Chieh Yu, Masatsugu Yokota, Goro Kokubugata

**Affiliations:** 1 Division of Forest and Biomaterials Science, Graduate School of Agriculture, Kyoto University, Kitashirakawa Oiwake-cho, Sakyo-ku, Kyoto, 606-8502, Japan National Museum of Nature and Science Ibaraki Japan; 2 Department of Botany, National Museum of Nature and Science, Amakubo 4-1-1, Tsukuba, Ibaraki 305-0005, Japan Kyoto University Kyoto Japan; 3 CAS Key Laboratory of Tropical Forest Ecology, Xishuangbanna Tropical Botanical Garden, Chinese Academy of Sciences, Menglun, Mengla, Yunnan 666303, China Xishuangbanna Tropical Botanical Garden, Chinese Academy of Sciences Yunnan China; 4 Laboratory of Ecology and Systematics, Faculty of Science, University of the Ryukyus, Senbaru 1, Nishihara, Okinawa 903-0213, Japan University of the Ryukyus Okinawa Japan

**Keywords:** Miyako Islands, phylogeny, stone crop, succulent plants, taxonomy

## Abstract

We re-examined the taxonomic status of plants treated as *Sedum formosanum* (Crassulaceae) from Miyako-jima Island of the Ryukyu Islands, Japan, using morphological comparison and molecular phylogenetic analyses with related species. In morphology, plants from Miyako-jima Island bore a close resemblance to the other plants of *S. formosanum*, but differed in being perennial, polycarpic, and having lateral axillary branches. Molecular analyses based on ITS of nrDNA and six regions of cpDNA sequencing indicated that the Miyako-jima plants formed a distinct subclade. This subclade was part of a polytomy with three other subclades comprising nine taxa endemic to Taiwan and *S. formosanum* from other areas, including the type locality. Therefore, we propose and describe the Miyako-jima plants as a new subspecies, Sedum formosanum subsp. miyakojimense.

## Introduction

The genus *Sedum* L. (Crassulaceae) comprises about 470 succulent herbaceous species (Thiede and Eggli 2007). Species within this genus are widely distributed in the Northern Hemisphere, and are most diverse in the Mediterranean Sea, Central America, the Himalayas, and East Asia (Stephenson 1994; Thiede and Eggli 2007). A previous phylogenetic study indicated that *Sedum* is a polyphyletic group within seven American genera (Carrillo-Reyes et al. 2009). However, in East Asia, *Sedum* has been shown to be monophyletic (Mayuzumi and Ohba 2004; Carrillo-Reyes et al. 2009). The Flora of China (Fu and Ohba 2001) divides East Asian *Sedum* species into three sections (sects.); *Sedum*, *Oreades* (Fröderström) K.T. Fu, and *Filipes* (Fröderström) S.H. Fu. Section Sedum is distinguished from sects. *Oreades* and *Filipes* by adaxially gibbous carpels and follicles, and sect. Oreades is differentiated from sect. Filipes by the absence of spurred leaves at the base. Additionally, species of sect. Oreades generally have yellow or purple-red (rarely red) petals, whereas members of sect. Filipes have white or reddish purple (rarely yellow) petals (Fu and Ohba 2001). Seventeen species of *Sedum* are reported from Japan, including four subspecies and four varieties within sect. Sedum, and one species within sect. Filipes (Ohba 2001).

**Figure 1.**
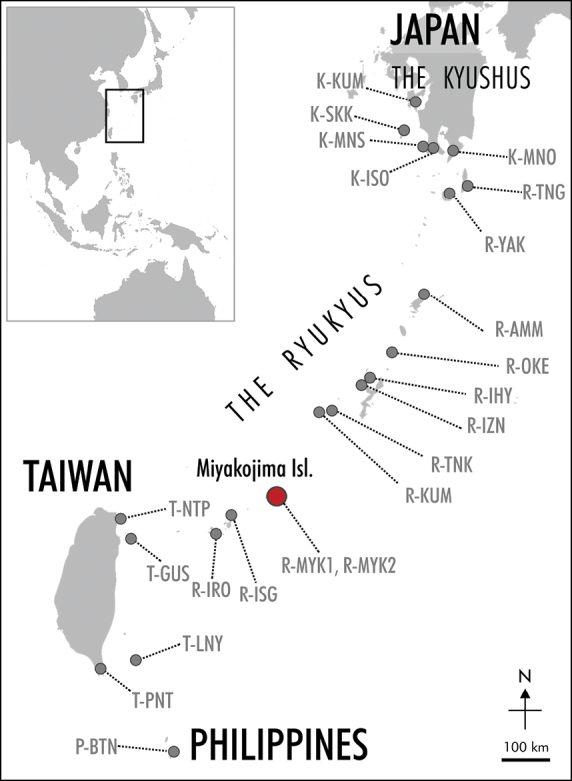
Map showing the location of Miyakojima Island and the adjacent area. The red circle indicates location of Miyako-jima Island. The gray circle indicates the others sample localities of *S. formosanum* (see Table 2 for abbreviations for collection localities).

*Sedum formosanum* N. E. Brown, described based on a type specimen collected from Taiwan (Brown 1885), occurs on rocky seashore slopes in the southern part of Kyushu in the Ryukyu Islands of Japan, in Taiwan, and on Batan Island in the Philippines (Hatusima 1975; Lin 1999; Ohba 2001; Hotta 2013; Shiuchi and Hotta 2015; Ryukyu Plant Research Group 2018). *Sedum formosanum*, a monocarpic biennial herb, is one of the few species of East Asian *Sedum* characterized by a trichotomous branching form (Ohba 2001). In Japan, populations of *S. formosanum* are scattered on the Ryukyu Islands (the Ryukyus), which comprise approximately 140 islands in a 1,300-km-long stretch between Kyushu and Taiwan (Fig. 1). Owing to its scarcity, this species is classified as ‘Near Threatened’ (NT) on the Red List of Threatened Species of Japan (Japanese Ministry of the Environment 2019). However, accurate identification of *Sedum* species can be hindered by high morphological similarity and plasticity. Therefore, there is a lack of clarity in the taxonomic identity of *S. formosanum* (Ito et al. 2017a). In fact, *Sedum* plants distributed on the Danjo Islands, Japan, which had historically been treated as *S. formosanum*, were recently described as a distinct taxon, *S. danjoense* Takuro Ito, H. Nakanishi & G. Kokub. (Ito et al. 2017a).

Based on previous field surveys, we noted that plants treated as *S. formosanum* on Miyako-jima of the Ryukyus differed morphologically from other populations. In this study, we conducted morphological comparisons and molecular phylogenetic analyses to elucidate the taxonomic status of plants treated as *S. formosanum* on Miyako-jima Island.

## Materials and methods

### DNA Sample collection

The plants treated as *S. formosanum* are only known from one locality on Miyako-jima Island. We collected two individuals of the plants from the island for DNA samples. To clarify the phylogenetic position of *S. formosanum* growing on Miyako-jima Island, we utilized ITS (Internal Transcribed Spacer region of nuclear ribosomal DNA) sequences of 50 taxa (72 accessions) of *Sedum* in Asia including *S. formosanum* from 20 localities in Kyushu, the Ryukyus, Taiwan and the Philippines as ingroup reported by previous study (Mayuzumi and Ohba 2004; Ito et al. 2014, 2017a, 2017b) (Tables 1, 2). Additionally, we sequenced one species of the eastern Asian species, *S. emarginatum* (Table 1). Following previously reported phylogenetic study of Crassulaceae (Mayuzumi and Ohba 2004), *Aeonium castello-paivae* Bolle, *A. gomerense* Praeger, *A. lancerottense* Praeger, *A. viscatum* Bolle, and *Greenovia aizoon* Bolle, which were collected by Mort et al. (2002) and stored in GenBank were selected as outgroups (Table 1). In total, 80 operational taxonomic units (OTUs) were included in our molecular phylogenetic analysis based on ITS (Tables 1, 2). Subsequently, we conducted molecular phylogenetic analysis based on six cpDNA (Chloroplast DNA) regions with *S. formosanum* and its close relatives to clarify the detailed phylogenetic relationships. Following Ito et al. (2017b), nine Taiwanese taxa were selected as ingroup, *S. alfredii* Hance, and *S. sekiteiense* Yamam. and *S. tricarpum* Makino were selected as outgroups (Table 3). In total, 27 OTUs were included in our molecular phylogenetic analysis based on cpDNA (Tables 2, 3). Taxonomic treatments tentatively followed Ohba (2001) and Ito et al. (2018) for Japanese taxa, Lin (1999) and Lu et al. (2019) for Taiwanese taxa, and Fu and Ohba (2001) for Chinese taxa. Voucher specimens for our collections were primarily deposited in the herbarium of the National Museum of Nature and Science, Japan (TNS).

**Table 1.** Plant materials of 53 accessions of eastern Asian *Sedum* taxa and five outgroup taxa with their collection locality, voucher information, and accession numbers of ITS sequences.

Taxon	Locality	Voucher (Herbarium)	Accession No.	Taxon	Locality	Voucher (Herbarium)	Accession No.
*S. actinocarpum*	Taiwan	*TI 1749* (TNS)	^1^ LC229265	S. polytrichoides ssp. polytrichoides	Japan	*TI 2247* (TNS)	^1^ LC229252
*S. alfredii*	China	*GK 17190* (IBSC)	^2^ AB930259	S. polytrichoides ssp. yabeanum var. yabeanum	Japan	*TI 396* (TNS)	^2^ AB906490
*S. arisanense*	Taiwan	*TI 1836* (TNS)	^1^ LC229272	S. polytrichoides ssp. yabeanum var. setouchiense	Japan	*TI 2298* (TNS)	^1^ LC229253
*S. boninense*	Japan	*TI 2371* (TNS)	^1^ LC229242	*S. rupifragum*	Japan	*TI 2070* (TNS)	^1^ LC229254
*S. bulbiferum*	Japan	*TI 416* (TNS)	^1^ LC229234	*S. sarmentosum*	China	*TI 978* (TNS)	^1^ LC229255
*S. brachyrinchum*	Taiwan	*TI 3118* (TNS)	^1^ LC229277	*S. satumense*	Japan	*TI 2295* (TNS)	^1^ LC229256
*S. danjoense*	Japan	*TI 3658* (TNS)	^3^ LC260127	*S. sekiteiense*	Taiwan	*TI 1456* (TNS)	^1^ LC229295
*S. emarginatum*	China	*TI 1062* (TNS)	LC530833	*S. subtile*	Japan	*TI 2259* (TNS)	^1^ LC229257
*S. erici-magnusii*	China	*TI 2077* (TNS)	^1^ LC229235	*S. taiwanalpinum*	Taiwan	*TI 1823* (TNS)	^1^ LC229278
*S. hakonense*	Japan	*TI 623* (TNS)	^2^ AB930278	*S. taiwanianum*	Taiwan	*TI 2523* (TNS)	^1^ LC229296
*S. hangzhouense*	China	*TI 2604* (TNS)	^3^ LC260130	*S. tarokoense*	Taiwan.	*TI 2025* (TNS)	^1^ LC229298
S. japonicum ssp. japonicum var. japonicum	Japan	*TI 723* (TNS)	^1^ LC229237	*S. tetractinum*	China	*TI 3623* (TNS)	^3^ LC260135
S. japonicum ssp. japonicum var. senanense	Japan	*TI 2200* (TNS)	^1^ LC229238	*S. tianmushanense*	China	*LP 67* (TNS)	^1^ LC229261
S. japonicum ssp. oryzifolium var. oryzifolium	Japan	*TI 2285* (TNS)	^1^ LC229239	*S. tosaense*	Japan	*TI 655* (TNS)	^1^ LC229258
S. japonicum ssp. oryzifolium var. pumilum	Japan.	*TI 2287* (TNS)	^1^ LC229240	*S. triactina*	Nepal	*TI 9596091* (TI)	^4^ AB088629
*S. jiulungshanense*	China	*CMQ 76* (TNS)	^1^ LC229243	*S. triangulosepalum*	Taiwan	*TI 2508* (TNS)	^1^ LC229299
*S. kiangnanense*	China	*TI 1030* (TNS)	^1^ LC229244	*S. tricarpum*	Japan	*TI 2269* (TNS)	^1^ LC229259
*S. kwanwuense*	Taiwan	*TI 2440* (TNS)	^1^ LC229293		China	*TI 3597* (TNS)	^3^ LC260134
*S. lineare*	Japan	*HU 667* (TNS)	^1^ LC229245	*S. trullipetalum*	Nepal	*TI 9420132* (TI)	^4^ AB088630
*S. lungtsuanense*	China	*TI 3563* (TNS)	^3^ LC260131	*S. truncastigmum*	Taiwan	*TI 2766* (TNS)	^1^ LC229305
*S. makinoi*	Japan	*TI 2325* (TNS)	^1^ LC229246	*S. uniflorum*	Japan	*TI 447* (TNS)	^1^ LC229241
*S. mexicanum*	Japan	*TI 647* (TNS)	^1^ LC229247	*S. zentaro-tashiroi*	Japan	*TI 355* (TNS)	^2^ AB906491
*S. microsepalum*	Taiwan	*TI 2771* (TNS)	^1^ LC229282	*Sedum* sp.	China	*JP 404* (TNS)	^1^ LC229262
*S. morrisonense*	Taiwan	*TI 2348* (TNS)	^1^ LC229289				
*S. multicaule*	China	*TI 625* (TNS)	^1^ LC229248	**Outgroup**			
*S. nokoense*	Taiwan	*TI 3196* (TNS)	^1^ LC229294	*Aeonium castello-paivae*	Canary	*MEM 1519* (WS)	^5^ AY082236
*S. nagasakianum*	Japan	*TI 2064* (TNS)	^1^ LC229249	*Aeonium gomerense*	Canary	*MEM 1454* (WS)	^5^ AY082242
*S. oligospermum*	China	*CMQ 74* (TNS)	^1^ LC229250	*Aeonium lancerottense*	Canary	*MEM 1518* (WS)	^5^ AY082143
*S. oreades*	Nepal	*TI 9420140* (TI)	^4^ AB088632	*Aeonium viscatum*	Canary	*MEM 1432* (WS)	^5^ AY082154
S. polytrichoides ssp. polytrichoides	China	*TI 1057* (TNS)	^1^ LC229251	*Greenovia aizoon*	Canary	*MEM 1425* (WS)	^5^ AY082112

Reported by ^1^Ito et al. (2017b), ^2^Ito et al. (2014), ^3^Ito et al. (2017a), ^4^Mayuzumi and Ohba (2004) and ^5^Mort et al. (2002).

**Table 2.** Plant materials of *Sedum formosanum* with their collection locality, voucher information, and accession numbers of ITS and cpDNA sequences.

Abbreviation	Locality	Voucher (Herbarium)	nrDNA	cpDNA					
			ITS	*mat*K-*trn*K	*ndh*A	*psb*M-*ycf*6	*rp*S16	*trn*D-*psb*M	trnL-F
P-BTN	Philippines: Batanes, Batan Isl.	*GK 15715* (TNS)	^1^ AB930273	^3^ LC258201	^3^ LC229400	^3^ LC258337	^3^ LC229468	^3^ LC258269	^3^ LC229536
K-MNS	Japan: Kyushu, Minami-satsuma.	*GK 16768* (TNS)	^1^ AB930262	^3^ LC258200	^3^ LC229399	^3^ LC258336	^3^ LC229467	^3^ LC258268	^3^ LC229535
K-MNO	Japan: Kyushu, Kagoshima, Minami-Osumi.	*TI 3238* (TNS)	^2^ LC260123	–	–	–	–	–	–
K-ISO	Japan: Kyushu, Kagoshima, Mt. Isoma.	*TI 2296* (TNS)	^2^ LC260124	–	–	–	–	–	–
K-SKK	Japan: Kyushu, Kagoshima, Shimo-Koshiki Isl.	*TI 3200* (TNS)	^2^ LC260125	–	–	–	–	–	–
K-KUM	Japan: Kyushu, Kumamoto, Reihoku.	*TI 637* (TNS)	^2^ LC260126	–	–	–	–	–	–
R-IHY	Japan: Ryukyus, Iheya Isl.	*GK 10726* (TNS)	^1^ AB930267	^3^ LC258193	^3^ LC229392	^3^ LC258329	^3^ LC229460	^3^ LC258261	^3^ LC229528
R-ISG	Japan: Ryukyus, Ishigaki Isl.	*GK 11775* (TNS)	^1^ AB906474	^3^ LC258194	^3^ LC229393	^3^ LC258330	^3^ LC229461	^3^ LC258262	^3^ LC229529
R-IZN	Japan: Ryukyus, Izena Isl.	*GK 12224* (TNS)	^1^ AB930266	^3^ LC258195	^3^ LC229394	^3^ LC258331	^3^ LC229462	^3^ LC258263	^3^ LC229530
R-KUM	Japan: Ryukyus, Kume Isl.	*GK 12755* (TNS)	^1^ AB930269	^3^ LC258196	^3^ LC229395	^3^ LC258332	^3^ LC229463	^3^ LC258264	^3^ LC229531
R-TNK	Japan: Ryukyus, Tonaki Isl.	*GK 13049* (TNS)	^1^ AB930268	^3^ LC258197	^3^ LC229396	^3^ LC258333	^3^ LC229464	^3^ LC258265	^3^ LC229532
R-TNG	Japan: Ryukyus, Tanegashima Isl.	*GK 15602* (TNS)	^1^ AB930265	^3^ LC258198	^3^ LC229397	^3^ LC258334	^3^ LC229465	^3^ LC258266	^3^ LC229533
R-AMM	Japan: Ryukyus, Amami Isl.	*GK 16712* (TNS)	^1^ AB930264	^3^ LC258199	^3^ LC229398	^3^ LC258335	^3^ LC229466	^3^ LC258267	^3^ LC229534
R-IRO	Japan: Ryukyus, Iriomote Isl.	*TI 598* (TNS)	^1^ AB930270	–	–	–	–	–	–
R-MYK1	Japan: Ryukyus, Miyako-jima Isl.	*TI 1115* (TNS)	LC530813	LC530834	LC530836	LC530838	LC530840	LC530842	LC530844
R-MYK2	Japan: Ryukyus, Miyako-jima Isl.	*TI 1120* (TNS)	LC530814	LC530835	LC530837	LC530839	LC530841	LC530843	LC530845
R-OKE	Japan: Ryukyu, Kagoshima, Okinoerabu Isl.	*TI 2611* (TNS)	^2^ LC260128	–	–	–	–	–	–
R-YAK	Japan: Ryukyu, kagoshima, Yakushima Isl.	*TI 2648* (TNS)	^2^ LC260129	–	–	–	–	–	–
T-LNY	Taiwan: Lanyu, Lanyu Isl.	*GK 6132* (TNS)	^1^ AB930271	^3^ LC258202	^3^ LC229401	^3^ LC258338	^3^ LC229469	^3^ LC258270	^3^ LC229537
T-NTP	Taiwan: New Taipei.	*GK 16446* (TNS)	^1^ AB930272	^3^ LC258203	^3^ LC229402	^3^ LC258339	^3^ LC229470	^3^ LC258271	^3^ LC229538
T-GUS	Taiwan: Yilan, Gueishan Isl.	*TI 1260* (TNS)	^3^ LC229279	^3^ LC258204	^3^ LC229403	^3^ LC258340	^3^ LC229471	^3^ LC258272	^3^ LC229539
T-PNT	Taiwan: Pingtung, Sheding.	*TI 1921* (TNS)	^3^ LC229280	^3^ LC258205	^3^ LC229404	^3^ LC258341	^3^ LC229472	^3^ LC258273	^3^ LC229540

Reported by ^1^Ito et al. (2014), ^2^Ito et al. (2017a), ^3^Ito et al. (2017b).

**Table 3.** Plant materials of Nine Taiwanese *Sedum* species and three outgroups which are closely relatives of *S. formosanum* with their collection locality, voucher information, and accession numbers of cpDNA sequences reported by Ito et al. (2017b).

Taxon	Locality	Voucher (Herbarium)	cpDNA					
			*mat*K-*trn*K	*ndh*A	*psb*M-*ycf*6	*rpS*16	*trn*D-*psb*M	*trn*L-F
*S. actinocarpum*	Taiwan	*TI 1749* (TNS)	LC258179	LC229378	LC258315	LC229446	LC258247	LC229514
*S. arisanense*	Taiwan	*TI 1836* (TNS)	LC258186	LC229385	LC258322	LC229453	LC258254	LC229521
*S. brachyrinchum*	Taiwan	*TI 3118* (TNS)	LC258191	LC229390	LC258327	LC229458	LC258259	LC229526
*S. kwanwuense*	Taiwan	*TI 2440* (TNS)	LC258218	LC229417	LC258354	LC229485	LC258286	LC229553
*S. microsepalum*	Taiwan	*TI 2771* (TNS)	LC258207	LC229406	LC258343	LC229474	LC258275	LC229542
*S. nokoense*	Taiwan	*TI 3196* (TNS)	LC258219	LC229418	LC258355	LC229486	LC258287	LC229554
*S. taiwanalpinum*	Taiwan	*TI1823* (TNS)	LC258192	LC229391	LC258328	LC229459	LC258260	LC229527
*S. tarokoense*	Taiwan	*TI2025* (TNS)	LC258223	LC229422	LC258359	LC229490	LC258291	LC229558
*S. triangulosepalum*	Taiwan	*TI2508* (TNS)	LC258224	LC229423	LC258360	LC229491	LC258292	LC229559
**Outgroup**								
*S. alfredii*	China	*GK 17190* (TNS)	LC258164	LC229363	LC258300	LC229431	LC258232	LC229499
*S. sekiteiense*	Taiwan	*TI1456* (TNS)	LC258220	LC229419	LC258356	LC229487	LC258288	LC229555
*S. tricarpum*	Japan	*TI2269* (TNS)	LC258175	LC229374	LC258311	LC229442	LC258243	LC229510

### DNA extraction, PCR amplification, and sequencing

DNA was extracted from dried leaves using a DNeasy Plant Mini Kit (Qiagen, Valencia, CA), in accordance with the manufacturer’s protocols. The ITS region containing the ITS1, 5.8S rDNA, and ITS2 and six regions of cpDNA (*mat*K-*trn*K, *ndh*A, *psb*M-*ycf*6, rpS16, *trn*D-*psb*M and *trn*L-F) sequences were amplified by polymerase chain reaction (PCR) with an iCycler (Bio-Rad, Hercules, CA, USA). The ITS and six regions of cpDNA sequences were amplified using EmeraldAmp PCR Master Mix dye (Takara, Otsu, Japan) and the following forward and reverse primers, respectively: ITS, primers ITS1 and ITS4 (White et al. 1990); *mat*K-*trn*K intron primers *mat*KAF and *trn*K2R; *ndh*A intron, primers *ndh*×1 and *ndh*×2 (Shaw et al. 2007); *psb*M-*ycf*6 intron, primers *psb*MR and *ycf*6F; *rpS*16 intron, primers *rpS*16F and *rpS*16R; *trn*D-*psb*M intron, primers *psb*MF and *trn*D (Shaw et al., 2005); and *trn*L-F, primers *trn*Lc and *trn*Ff (Taberlet et al. 1991) by an iCycler (Bio-Rad, Hercules, CA). The PCR profile consisted of an initial 3 min at 94°C followed by 35 cycles of 30 s at 94°C, 30 s at 50°C for the ITS sequence or 55°C for the cpDNA sequence, and 90 s at 72°C. The PCR product were purified by ExoStar clean-up kit (USB, Cleveland, OH). Cycle sequencing was performed using a BigDye Terminator Cycle Sequencing Kit ver. 3.1 (Applied Biosystems, Foster City, CA) and the PCR primers mentioned above for the ITS and cpDNA sequences. The Sanger sequencing products were then purified by ethanol precipitation. Automated sequencing was carried out with an Applied Biosystems 3130xl Genetic Analyzer. The electropherograms were assembled using ATGC ver. 6 (GENETYX, Tokyo, Japan). The sequence data obtained in this study were deposited in the DDBJ/EMBL/GenBank database (http://www.ncbi.nlm.nih.gov/gquery/).

### Phylogenetic analysis using ITS and cpDNA sequences

The ITS and cpDNA sequences were aligned using ClustalW 1.8 (Thompson et al. 1994) and then adjusted manually. Phylogenetic analyses were conducted with a Bayesian approach using MrBayes 3.1.2 (Ronquist and Huelsenbeck 2003) and maximum-likelihood (ML) phylogenetic analysis using RAxML (Stamatakis 2014). In the Bayesian phylogenetic analysis, we used Akaike’s Information Criterion (AIC) implemented in MrModeltest 2.2 (Nylander 2004) to obtain an appropriate evolutionary model of nucleotide substitutions. And then we performed two separate runs of Metropolis-coupled Markov chain Monte Carlo (MCMCMC) analysis, each with a random starting tree and four chains (one cold and three hot) based on the selected model. The MCMCMC length was one million generations, and the chain was sampled every one hundredth generation from the cold chain. The first 2,500 sample trees (25% of the total 10,000 sample trees) were discarded as burn-in after checking that the average standard deviation of split frequencies (ASDSF) reached a stationary state at < 0.01 thereafter. A 50% majority consensus tree of the output tree file from MrBayes was generated using FigTree ver. 1.3.1 (Rambaut 2009). The ML phylogenetic analyses were implemented in RAxML 8 (Stamatakis 2014) with a GTRGAMMA substitution model. The ML bootstrap proportions (BPs) and trees were obtained by simultaneously running rapid bootstrapping with 1,000 iterations followed by a search for the most likely tree.

## Intraspecific morphological comparison

The plants known as *S. formosanum* from Miyako-jima Island (*T. Ito 1115*, *1120*, *2402* and *2408*, TNS) were used for morphological comparisons. Herbarium specimens of *S. formosanum* deposited in the Kagoshima University Museum (KAG), the University of the Ryukyus (RYU), the National Museum of Nature and Science (TNS), the National Taiwan University (TAI) and the Taiwan Forestry Research Institute (TAIF) were examined. By field survey, the phenotypic plasticity of leaf shape in response to environmental changes was observed. Therefore, we also have cultivated the plants from Miyako-jima Island and from Taiwan, where the type locality of the species is, in Tsukuba Botanical Garden to compare their leaf shape and life cycle during 2015–2017.

## Results and discussion

### Phylogenetic analyses using ITS and cpDNA

**Figure 2.**
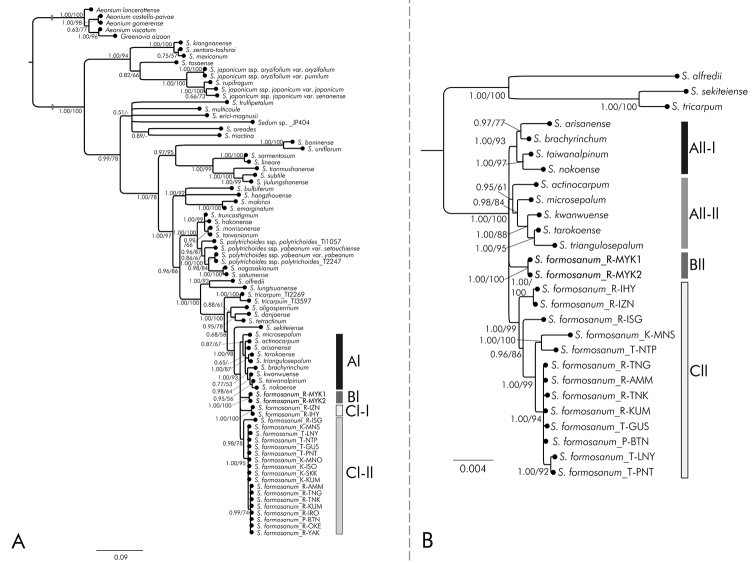
Bayesian phylogenetic tree based on ITS sequences for Eastern Asian *Sedum* (**A**), and Bayesian phylogenetic tree based on cpDNA for sequences *S. formosanum* and its closely relatives (**B**). The topology of the maximum likelihood (ML) tree was highly compatible with the Bayesian tree. Bayesian posterior probabilities (PPs: left) and bootstrap percentages from ML analysis (BP: right) are shown (See Tables 1, 2 and 3 for the abbreviations of localities).

We used 80 operational taxonomic units (OTUs), including 75 as ingroup accessions and 5 as outgroup accessions in the Bayesian and ML analyses based on ITS sequences (Tables 1, 2). Following alignment, we obtained a matrix of 629 base pairs (bp) and selected GTR+I+G for the Bayesian analysis. The 50% majority rule consensus tree of all post burn-in trees is shown with Bayesian posterior probabilities (PPs) in Fig. 2A. The topology of the ML tree was highly compatible with that of the Bayesian tree (Fig. 2A). In both the Bayesian and ML analyses based on ITS sequences, *S. formosanum* and nine taxa endemic to Taiwan formed a well-supported clade (PP/BS = 1.00/93). Within this clade, four subclades that formed a polytomy were recognized: nine taxa endemic to Taiwan (0.87/67, Clade Al), *S. formosanum* from Miyako-jima Island (1.00/100, Clade Bl), *S. formosanum* from Izena Island and Iheya Island (1.00/100, Clade Cl-l), and *S. formosanum* from 18 accessions from Japan (excluding Miyako-jima Island, Iheya Island, and Izena Island), Taiwan, and the Philippines (0.98/78, Clade Cl-ll).

We used 29 OTUs, including 26 accessions as ingroups and 3 as outgroups in the Bayesian and ML analyses based on combined six regions of cpDNA sequence (Tables 2, 3). Following alignment, we obtained a matrix of 5,115 bp. In the resulting Bayesian and ML phylogenetic trees, we observed a topology similar to the trees formed using ITS data. We again observed strong evidence that *S. formosanum* and nine taxa endemic to Taiwan formed a well-supported clade with four subclades (1.00/100; Fig. 2B). However, these four subclades formed a polytomy that differed from that suggested by the ITS tree. Although *S. formosanum* from Miyako-jima Island was again supported as forming a subclade (1.00/100, Clade Bll), we found that the nine Taiwanese endemics were divided into two subclades (1.00/93, Clade All-l; 0.95/61, Clade All-ll), and *S. formosanum* on Izena Island and Iheya Island formed a subclade with the 18 accessions from Japan (excluding Miyako-jima Island), Taiwan and the Philippines (1.00/99, Clade Cll).

### Morphological comparison

We observed a similar flower morphology among the herbarium specimens from Miyako-jima Island (TNS; *T. Ito 1115*, *1120*, *2402*, and *2408*) and those from other regions in Japan, Taiwan, and the Philippines. Generally, *S. formosanum* displays trichotomous branching at the shoot tip and does not produce lateral branches. The Miyako-jima plants also displayed trichotomous branching at the shoot tips, but they often developed lateral branches in the leaf axils of long shoots. Additionally, we found similar plants of *S. formosanum* that also produce axillary lateral branches on Ishigaki Island, part of the Yaeyama Islands, on Gaja-jima Island and Akuseki-jima Island in the Tokara Islands, and on Yoron Island in the Amami Islands by specimen survey.

In terms of leaf morphology, we observed high variation and no clear difference between the Miyako-jima plants and those from other locations. To remove the potentially confounding influence of environmental factors on leaf morphology, we cultivated plants from both Miyako-jima Island and Taiwan (obtained from the type locality) and compared them. Using this approach, we detected slight differences in leaf shape. Plants from Miyako-jima Island had spatulate to oblanceolate leaves, whereas plants from Taiwan had leaves that were spatulate to widely obovate. Most notably, plants from Miyako-jima Island were perennial and polycarpic, whereas plants from Taiwan were biennial and monocarpic.

### Intraspecific taxonomy of *S. formosanum*

**Figure 3.**
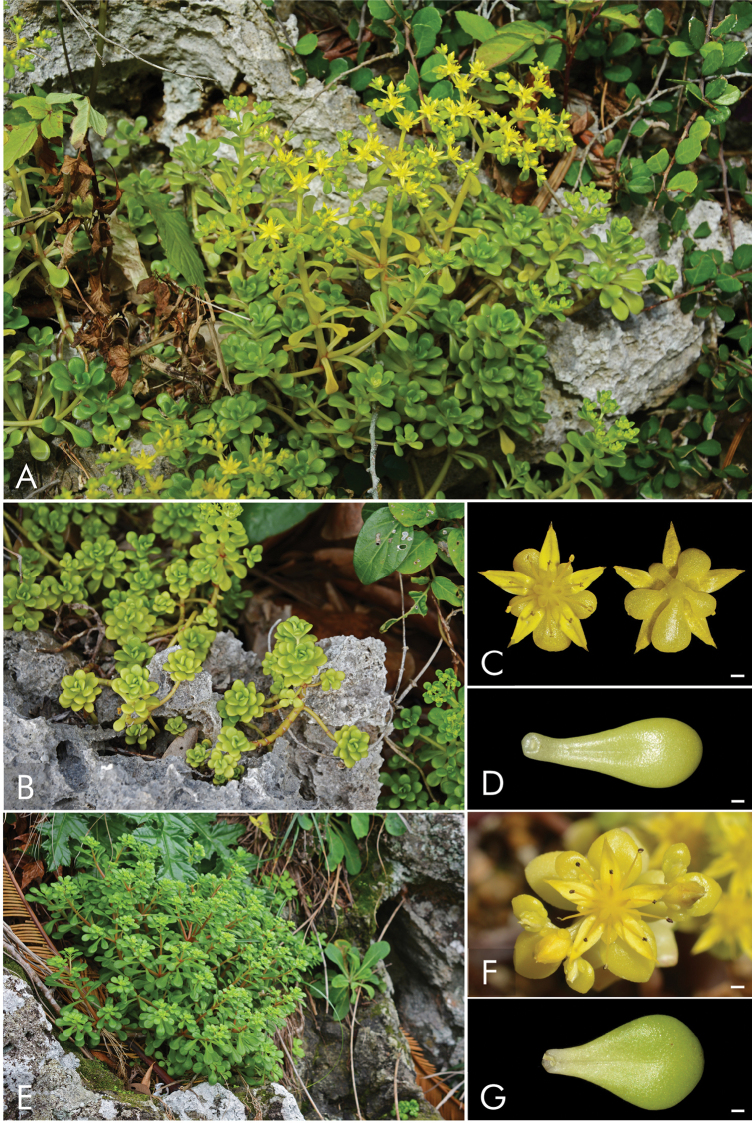
Sedum formosanum subsp. miyakojimense (**A–D***T. Ito 2402*, *2408*, Miyako-jima Island of the Ryukyus, Japan) and S. formosanum subsp. formosanum (**E** Kume-jima Island of the Ryukyus, Japan. **F, G** NewTaipei City, Taiwan). **A, B, E** habit **C, F** flower **D, G** leaf. Scale bars: 1 mm (**D–F**).

**Figure 4.**
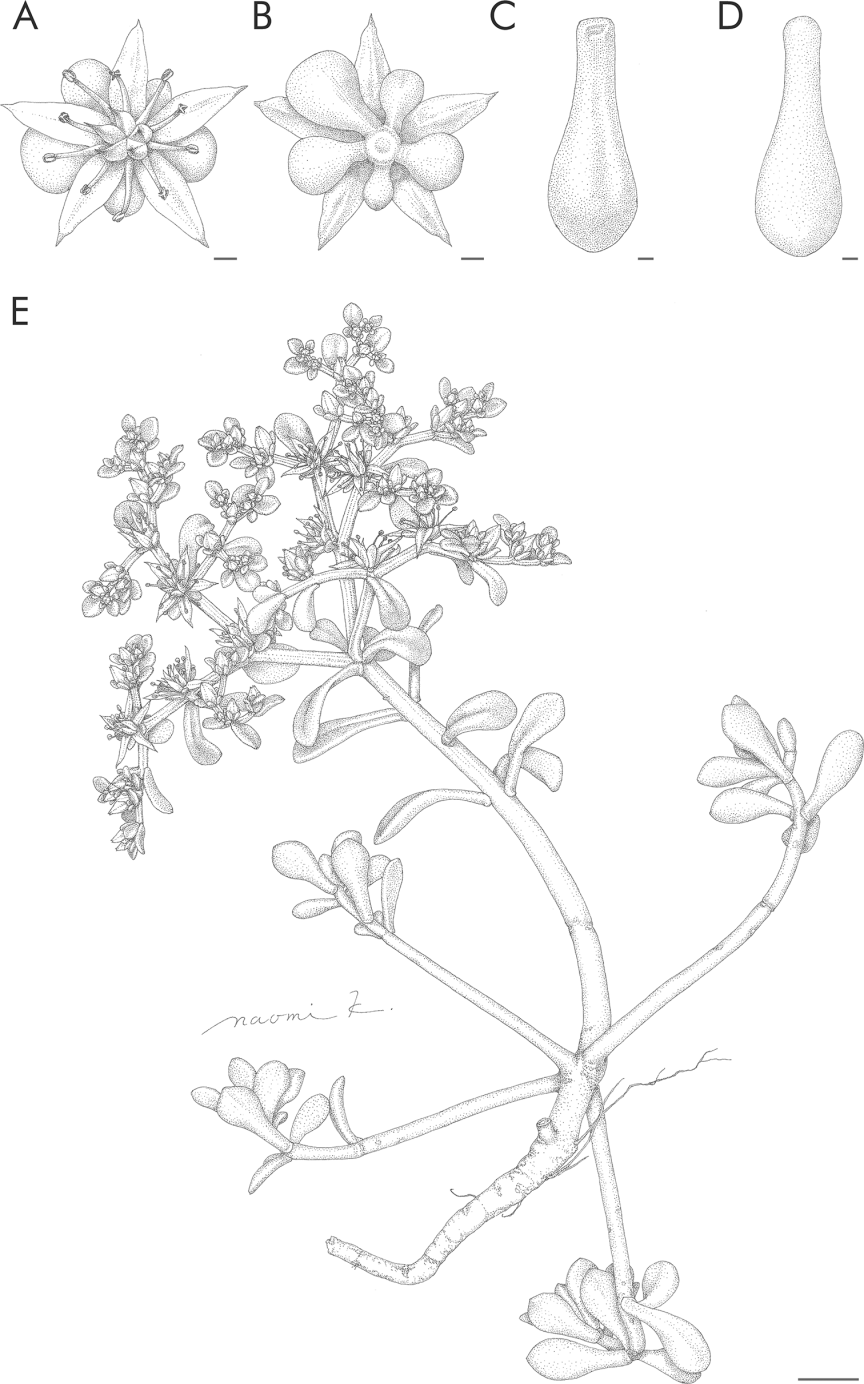
Sedum formosanum subsp. miyakojimense. **A** flower **B** sepal **C** leaf, adaxial **D** leaf, abaxial **E** habit. Scale bars: 1 mm (**A–D**); 1 cm (**E**). Line drawings by Naomi Kizaki.

The molecular phylogenetic analyses based on both ITS and cpDNA indicated that the *Sedum* species from Miyako-jima Island, which are currently considered as *S. formosanum*, formed a well-supported clade. This clade was distinct from that of *S. formosanum* collected from other regions of Japan, Taiwan (including the type locality), and the Philippines (Fig. 2). Morphologically, plants from Miyako-jima Island were distinguishable from plants from other areas due to the presence of axillary lateral branches and by life cycle, i.e., perennial and polycarpic versus biennial and monocarpic (Figs 3, 4). Leaf shape differed slightly between the Miyako-jima plants and those from other locations, i.e., spatulate to oblanceolate versus spatulate to widely obovate (Figs 3, 4). Therefore, we concluded that *S. formosanum* from Miyako-jima Island should be considered a distinct taxonomic entity and have thus described a new subspecies in this study.

Additionally, molecular phylogenetic trees based on both ITS and cpDNA suggested that *S. formosanum* on Iheya Island and Izena Island part of the Okinawa Islands formed a distinct clade (Fig. 2). Samples from Ishigaki Island in the Yaeyama Islands were also genetically distinct from the individuals from other islands (Fig. 2). However, no clear morphological differences could be observed between plants from Iheya Island and Izena Island and plants from Taiwan (including type locality). Plants from both Ishigaki Island and Miyako-jima Island had axillary lateral branches, however the life cycle and leaf morphology of the samples collected from Ishigaki Island were not in the focus of this study. Furthermore, plants from Akuseki Island, Gaja Island, and Yoron Island also have axillary lateral branches. However, we observed plants from all three islands developing flowering stems between August and October by specimen survey. Thus, they are likely autumn-flowering. Among Japanese *Sedum*, autumn-flowering is only reported in *S. danjoense*, which had been treated as *S. formosanum* and was described as an independent species recently (Ito et al. 2017a). Although the phylogenetic position of the populations on Akuseki Island, Gaja Island, and Yoron Island is uncertain, the plant may be closely related to *S. danjoense*. Therefore, further reconsideration of *S. formosanum* at the species and infraspecific level is needed to establish the circumscription of the species.

### Taxonomic treatment

#### Sedum formosanum N.E. Brown., subsp. formosanum

Taxon classificationPlantaeSaxifragalesCrassulaceae

5A56B63D-4D09-596C-AE0C-8C291365E620

Fig. 3E–G

≡ Sedum mariae Raym.-Hamet, Repert. Spec. Nov. Regni Veg. 8: 143. 1910. Type: Japan. Insula Oshima (Liukiu): Jul 1900, Faurie, U. J. 3923 (holotype: G [G00356298]).

##### Type.

Taiwan. Keelung City, date unknown, *C. Ford s.n.* (lectotype, designated by Byalt, V. V.: K [K000838648]; isotype, designated by N. E. Br. 1885, pg. 134: GH [GH00042587]).

##### Description.

Usually biennial herb, fleshy, glabrous. First year stem stout, erect, partly woody, 1 or 2 trifurcate, 3–10 cm tall, with lax rosettes; rosettes 3–18 cm wide with 15–45 leaves. Flowering stems fleshy, 10–30 cm tall, base ca. 5 mm broad, usually reddish or yellowish green, erect or sprawling and creeping at base, 1- or 2-trifurcate at base. Roots fibrous, sometimes adventitious at the leaf scar. Leaves alternate, evenly arranged, sessile, green or yellowish, flattish, ± thick, spatulate to widely obovate, 1.2–3.2 cm long, 0.5–1.6 cm wide, apex rounded, base long, attenuate, margins entire. Inflorescences terminal, cymes, 1 or 2 trifurcate with 3 (rarely 4) primary axes; primary axis 2–7 cm long, ascending, 1 to several times irregularly and often unequally forking, with a flower at each fork, ultimate branches 1–2 cm long, 3–6 flowered; bracts leaf-like, smaller than cauline leaves. Flowers 5 (rarely 6)-merous, 8–12 mm wide, sessile. Sepals 5, free, yellowish green, fleshy, flattish, unequal in size, obovate to oblanceolate, 2–4 mm long, 1.5–3 mm wide, apex round or obtuse, base spurred. Petals 5, bright yellow, lanceolate, 5–6 mm long, 1.3–1.6 mm wide, apex acuminate, base slightly connate. Stamens 10, shorter than petals, 4.8–5 mm long, erect at flowering, two-whorled arrangement; anthers oblong-lanceolate, ca. 0.5 mm long, deep yellow before dehiscence. Pistils 5, 5.5–6.5 mm long; carpels 5, free, connate at the base, gibbous ventrally. Fruits star-shaped, follicle, erect, 5.5–7 mm long. Flowering in April to June.

##### Distribution and habitat.

Japan: Kyushu, Kagoshima, Kumamoto; The Ryukyus, the Osumi Islands, Kami-Koshiki, Kuro-shima, Yaku-shima and Tanega-shima islands, the Tokara Islands, Akuseki, Gaja, Nakano-shima, Kodakara, Kuchino-shima, and Takara islands, the Amami Islands, Amami-oshima, Kakeroma, Kikai, Okierabu, Tokuno-shima, Uke, Yoro and Yoron islands, the Okinawa Islands, Aka, Geruma, Ie, Iheya, Izena, Kume, Okinawa, Sesoko, Tokashiki and Tonaki islands, the Yaeyama Islands, Ishigaki, Iriomote, Kuro-shima and Yonaguni islands. Taiwan: New Taipei, Keelung, Ilan, Hualien, Lienchiang, Taitung (Lanyu and Green Islands) and Pingtung. The Philippines: Batanes, Batan Island.

Coastal and rarely inland rocky slopes, xeric, saline, and exposed to direct sunlight.

##### Additional specimens examined.

Japan. Kyushu, Kagoshima: Ichikikushikino City, 30 June 1957, *S. Hatusima 20967 (KAG)*, Minamisatsuma City, 23 May 1962, *M. Furuse 325 (KAG)*, Minamisatsuma City, 9 Nov. 1984, *S. Sako 8865 (KAG)*, Minamisatsuma City, 14 Nov. 1987, S. *Hatusima 43027 (KAG)*, Ichikikushikino City, 26 June 2003, *K. Maruno s.n. (KAG)*, Minamisatsuma City, 23 June 2013, *G. Kokubugata, Y. Saito, T. Ito 16768 (TNS)*, Kimotsuki Country, Minamiosumi Town, 30 July 1949, *S. Hatusima 13352 (KAG)*, Kimotsuki Country, Minamiosumi Town, 13 June 1957, *S. Hatusima 20891 (KAG)*, Kimotsuki Country, Minamiosumi Town, 22 June 2013, G. *Kokubugata & T. Ito 16764 (TNS)*, Kimotsuki Country, Sata Village, 26 Aug. 1910, *Y. Nakano s.n. (TNS)*, Kimotsuki Country, Sata Village, 9 Aug. 1929, *H. Asuyama s.n. (TNS)*, Kimotsuki Country, Sata Town, 28 Mar. 1958, *S. Okuyama & H. Utsumi 17098 (TNS)*, Kumamoto: Amakusa Country, Reihoku Town, 13 Jan. 1956, *R. Moran 5395 (TNS)*, The Ryukyus, the Osumi Islands, Kagoshima: Kami-Koshiki Island, Satsuma Country, Kamikoshiki Village, 26 Mar. 1930, *K. Naohara s.n. (TNS)*, Kuro-shima Island, Kagoshima Country, Mishima Village, 12 June 1981, *K. Maruno s.n. (KAG)*, Kagoshima Country, Mishima Village, 26 May 1994, *T. Shiuchi 4900 (KAG)*, Tanega-shima Island, Nishinoomote City, 25 Feb. 2013, *G. Kokubugata, M. Yokota, K. Kaburagi 15604 (TNS)*, The Ryukyus, the Tokara Islands, Kagoshima: Akuseki Island, Kagoshima Country, Toshima Village, 18 Oct. 1980, *R. Yanagida s.n. (KAG)*, Kagoshima Country, Toshima Village, 9 Sep. 1983, *Y. Hukushima s.n. (KAG)*, Kagoshima Country, Toshima Village, 15 Oct. 1993, *T. Shiuchi 2800 (KAG)*, Gaja Island, Kagoshima Country, Toshima Village, 21 Aug. 1958, *S. Sako & K. Kawanabe 2244 (KAG)*, Nakano-shima Island, Kagoshima Country, Toshima Village, 18 Aug. 1958, *S. Sako & K. Kawanabe 1938 (KAG)*, Takara Island, Kagoshima Country, Toshima Village, 25 Aug. 1910, *S. Kawagoe s.n. (TNS)*, Kagoshima Country, Toshima Village, 11 Feb. 1952, *S. Hatusima s.n. (KAG)*, Kagoshima Country, Toshima Village, 14 May 1993, *T. Shiuchi 1314 (KAG)*, The Ryukyus, the Amami Islands, Kagoshima: Amami-oshima Island, Amami City, 28 Apr. 2012, *G. Kokubugata 16712 (TNS)*, Amami City, 26 Aug. 2014, *G. Kokubugata & H. Umemoto 18178 (TNS)*, Amami City, 12 Jan. 2016, *G. Kokubugata & M.Tabata 19011 (TNS)*, Naze City, 23 May 1975, *J. Haginiwa JH006639 (TNS)*, Naze City, 23 May 1975, *J. Haginiwa JH032447 (TNS)*, Naze City, 23 Nov. 1977, *A. Yamamoto, T. Nakaike & M. Ishizuka 490 (TNS)*, Oshima Country, Setouchi Town, 18 July 1919, *S. Kawagoe s.n. (KAG)*, Oshima Country, Setouchi Town, 6 Aug. 1956, *S. Ouchiyama 49 (KAG)*, Oshima Country, Setouchi Town, 24–28 July 1975, *Y. Miyagi & S. Hatusima 40407 (RYU)*, Oshima Country, Tatsugo Town, 27 Apr. 2012, *G. Kokubugata 16722 (TNS)*, Kakeroma Island, Oshima Country, Setouchi Town, 11 Jan. 2016, *G. Kokubugata, M. Tabata 18978 (TNS)*, Kikai Island, Oshima Country, Kikai Town, 17 May 1975, *K. Yoshinaga 178 (KAG)*, Okierabu Island, Oshima Country, China Town, 4 June 1967, *M. Furuse s.n. (KAG)*, Oshima Country, China Town, date unknown 1969, *K. Kasuga s.n. (KAG)*, Oshima Country, China Town, 7 Nov. 1971, *J. Haginiwa JH006572 (TNS)*, Tokuno-shima Island, Oshima Country, Amagi Town, 4 May 2014, *G. Kokubugata & H. Umemoto 17613 (TNS)*, Oshima Country, Tokunoshima Town, 3 May 2014, *G. Kokubugata & H. Umemoto 17556 (TNS)*, Uke Island, Oshima Country, Setouchi Town, 23 Mar. 2019, *E. Suzuki s.n. (KAG)*, Yoro Island, Oshima Country, Setouchi Town, 22 May 2018, *E. Suzuki s.n. (KAG)*, Oshima Country, Setouchi Town, 22 May 2018, *E. Suzuki s.n. (KAG)*, Yoron Island, Oshima Country, Yoron Town, 21 Aug. 1921, *K. Uyehara s.n. (KAG)*, Oshima Country, Yoron Town, 16 Aug. 1961, *G. Ikeda s.n. (KAG)*, Oshima Country, Yoron Town, 16 Aug. 1961, *G. Ikeda s.n. (KAG)*, Oshima Country, Yoron Town, 24 Dec. 1971, *J. Haginiwa JH006509 (TNS)*, Oshima Country, Yoron Town, 24 Dec. 1971, *J. Haginiwa JH006571 (TNS)*, The Ryukyus, the Okinawa Islands, Okinawa: Aka Island, Shimajiri Country, Zamami Village, 23–26 May 1974, *Y. Miyagi & T. Kabashima 4865 (RYU)*, Geruma Island, Shimajiri Country, Zamami Village, 9–12 Aug. 1977, *Y. Miyagi 7906 (RYU)*, Ie Island, Kunigami Country, Ie Village, 4–5 May. 1974, *S. Hatusima & Y. Miyagi 37591 (RYU)*, Kunigami Country, Ie Village, 16 Sep. 2014, *G. Kokubugata, M. Yokota et al. 18248 (TNS)*, Iheya Island, Shimajiri Country, Iheya Village, 25 Dec. 1958, *Y. Niiro s. n. (RYU)*, Shimajiri Country, Iheya Village, 26 May 2008, *G. Kokubugata 10726 (TNS)*, Izena Island, Shimajiri Country, Izena Village, 22 July 1973, *S. Hatusima 34901 (RYU)*, Shimajiri Country, Izena Village, 1 June 2015, *T. Yamada TYD263-1 (TNS)*, Kume Island, Shimajiri Country, Kumejima Town, 1 June 2010, *G. Kokubugata, M. Yokota & K. Nakamura 12755 (TNS)*, Okinawa Island, Itoman City, Aug. 1966, *Y. Miyagi 3636 (RYU)*, Itoman City, Aug. 1967, *Y. Miyagi 5654 (RYU)*, Itoman City, 7 May 2001, *G. Kokubugata & C.I. Peng 289 (TNS)*, Onna Village, 18 May 1980, *Y. Miyagi 9080 (RYU)*, Kunigami Country, Kunigami Village, May 1974, *S. Itoman 63 (RYU)*, Kunigami Country, Motobu Town, 3 May 1974, *S. Hatusima & Y. Miyagi 37633 (RYU)*, Nakagami Country, Kitanakagusuku Village, 30 Apr. 1955, *S. Hatusima 17462 (KAG)*, Nakagami Country, Kitanakagusuku Village, 30 Apr. 1955, *S. Hatusima 17498 (KAG)*, Shimajiri Country, Miwa Village, 23 May 1954, *S. Nakamine 68 (RYU)*, Shimajiri Country, Miwa Village, 23 May 1954, *S. Nakamine 68 (TNS)*, Sesoko Island, Kunigami Country, Motobu Town, 19 Aug. 1974, *Y. Miyagi 4202 (RYU)*, Tokashiki Island, Shimajiri Country, Tokashiki Village, 5 Mar. 1973, *Y. Miyagi & S. Oyadomari 1152 (RYU)*, Tonaki Island, Shimajiri Country, Tonaki Village, 10 Mar. 1973, *S. Hatusima 34404A (RYU)*, Shimajiri Country, Tonaki Village, 17 Dec. 2010, *G. Kokubugata & M. Yokota 13049 (TNS)*, The Ryukyus, the Yaeyama Islands, Okinawa: Ishigaki Island, Ishigaki City, 27 Mar. 2009, *G. Kokubugata, M. Yokota & K. Nakamura 11775 (TNS)*, Kuroshima Island, Yaeyama Country, Taketomi Town, 4 Nov. 1974, *Y. Niiro & Y. Miyagi 6103 (RYU)*, Yonaguni Island, Yaeyama Country, Yonaguni Town, 26–30 Oct. 1959, *S. Hatusima 24587 (KAG)*, Yaeyama Country, Yonaguni Town, 29 Sep. -3 Oct. 1973, *S. Hatusima, Y. Miyagi & E. Tanaka s.n. (TNS)*, Yaeyama Country, Yonaguni Town, 1 Nov. 1988, *R. Minagawa s.n. (TNS)*, Yaeyama Country, Yonaguni Town, 8 Dec. 2014, *G. Kokubugata, M. Yokota et al. 18586 (TNS)*, Yaeyama Country, Yonaguni Town, 7 Dec. 2014, *G. Kokubugata, M. Yokota et al. 18548 (TNS)*, Yaeyama Country, Yonaguni Town, 24 Nov. 2015, *T. Yamada TYD371 (TNS)*, TAIWAN. Hualien: Hualien City, 13 Dec. 1993, *T. C. Huang 15022 (TAI)*, Xiulin Township24 May 1993, *S. F. Huang, K. C. Yang & J. M. Hu 5097 (TAI)*, Ilan: Su’ao Township, 18 Apr. 1987, *S. F. Huang, C. F. Hsieh, Y. F. Lin et al. 3722 (TAI)*, Su’ao Township, 18 Apr. 1987, *W. S. Tang 1795 (TAI)*, Su’ao Township, 21 May 1987, *W. S. Tang 1803 (TAI)*, Su’ao Township, 18 Apr. 1987, *W. S. Tang 1785 (TAI)*, Su’ao Township, 21 May 1987, *W. S. Tang 1802 (TAI)*, Su’ao Township, 7 May 1993, *S. F. Huang 5075 (TAI)*, Su’ao Township, 21 May 1987, *W. S. Tang 1802 (TAI)*, Su’ao Township, 6 May 1993, *S. F. Huang 5049 (TAI)*, Kueishan Island, Toucheng Township, 31 May 1970, *C. C. Hsu 7237 (TAI)*, Toucheng Township, 3 July 1932, *G. Masamune & S. Suzuki s. n. (TAI)*, Keelung: Keelung City, 11 Oct. 2004, *S. W. Chung 7657 (TAIF)*, Keelung City, 7 June 2005, *S. W. Chung 7774 (TAIF)*, Keelung City, 6 June 2005, *P. F. Lu 9825 (TAIF)*, Keelung City, 23 May 2010, *P. F. Lu 20381 (TAIF)*, Keelung City, 12 July 2011, *P. F. Lu 22356 (TAIF)*, Keelung City, 22 July 1918, *M. Eizi 907 (TAI)*, Keelung City, 4 June 1932, *K. Mori s. n. (TAI)*, Keelung City, 31 May 1930, *S. Sasaki 4687 (TAI)*, Keelung City, 26 May 1939, *G. Masamune 1907 (TAI)*, Keelung City, 1 May 1937, *H. Simada 1218 (TAI)*, Keelung City, 3 June 1978, *C. M. Kou 9805 (TAI)*, Keelung City, 27 Apr. 1983, *C. L. Chang 91 (TAI)*, Keelung City, 1 May 1937, *H. Simada 1218 (TAI)*, Keelung City, date unknown, *M. L. Weng 66 (TAI)*, Pengchia Island, Keelung City, 4 Aug. 1992, *T. C. Huang 15753 (TAI)*, Lienchiang: Nangan Township, 29 June 1999, *S. H. Su s. n. (TAI)*, New Taipei: New Taipei City, 30 Apr. 2005, *P. F. Lu 9571 (TAIF)*, New Taipei City, 14 Aug. 2008, *Y. F. Chang s. n. (TAIF)*, New Taipei City, 6 June 1987, *W. S. Tang 1808 (TAI)*, New Taipei City, 6 June 1987, *W.S. Tang 1808 (TAI)*, New Taipei City, 23 Apr. 1929, *Y. Kudo, S. Suzuki & K. Mori 398 (TAI)*, New Taipei City, 23 Sep. 1931, *T. Tanaka s. n. (TAI)*, New Taipei City, 3 May 1986, *W. S. Tang 1757 (TAI)*, New Taipei City, 20 Apr. 1932, *T. Tanaka & Y.Simada 10963 (TAI)*, New Taipei City, 20 Apr. 1932, *T. Tanaka & Y.Simada s. n. (TAI)*, New Taipei City, 12 Apr. 1979, *S. H. Lin 692 (TAI)*, New Taipei City, 12 Apr. 1979, *C. M. Kuo 10939 (TAI)*, New Taipei City, 26 May 1985, *J. C. Wang 3349 (TAI)*, New Taipei City, 12 Apr. 1979, *H. N. Yang 2551 (TAI)*, New Taipei City, 27 May 1987, *W. S. Tang 1806 (TAI)*, New Taipei City, 27 May 1987, *W. S. Tang 1806 (TAI)*, New Taipei City, 20 Apr. 2002, *S. F. Cheng, S. K. Yu s. n. (TAI)*, New Taipei City, 31 May 2001, *Y. J. Lai, W. H. Wu et al. 745 (TAI)*, New Taipei City, 13 May 1988, *S. F. Huang 4297 (TAI)*, New Taipei City, 7 June 1930, *S. Sasaki 4744 (TAI)*, New Taipei City, 16 Apr. 1961, *T. C. Huang 2280 (TAI)*, New Taipei City, 7 June 1989, *W. S. Tang & C. F. Hsieh 1864 (TAI)*, New Taipei City, 30 May 1985, *T. Y. Yang 2020 (TAI)*, Pingtung: Hengchun Township, June 1912, *T. Kawakami & S. Sasaki s. n. (TAI)*, Taitung: Lanyu Island, Lanyu Township, 13 Jan. 1995, *T.P. Pan, C-H. Horng et al. s. n. (TAIF)*, Lanyu Township, 19 Mar. 1943, *T. Hosokawa 9896 (TAI)*, Lanyu Township, 17 Apr. 1992, S. F. *Huang & Y.C. Hsu 4735 (TAI)*, Lanyu Township, 29 Apr. 1983, *T.C. Huang, Yang, Kao et al. 9440 (TAI)*, Lanyu Township, 19 Feb. 1986, *T.C. Huang, S. F. Huang, K.C. Yang et al. 10535 (TAI)*, Lanyu Township, 17 Aug. 1958, *T. I. Chuang & C. C. Hsu 2384 (TAI)*, Lanyu Township, 18 Apr. 1932, *T. Sata 1286 (TAI)*, Lanyu Township, 0 May 1924, *S. Sasaki s. n. (TAI)*, Lanyu Township, 18 Apr. 1932, *T. Sata s. n. (TAI)*, Lanyu Township, 2 Apr. 1985, *S. F. Huang 2742 (TAI)*, Lanyu Township, 6 Apr. 1983, *T. C. Huang et al. 9205 (TAI)*, Lanyu Township, 6 Apr. 1983, *T. C. Huang et al. 9179 (TAI)*, Green Island, Lyudao Township, 4 Mar. 1931, *T.Tanaka 10373 (TAI)*, THE PHILIPPINES. The Batan Islands, Batanes: Batan Island, 9 Nov. 1964, *S. Hatusima & M. Sato 28624 (KAG)*.

#### Sedum formosanum N.E. Brown., subsp. miyakojimense

Taxon classificationPlantaeSaxifragalesCrassulaceae

Takuro Ito, Yokota & Kokub. subsp. nov.

37C59347-24F5-555D-89A9-9F274E8F30F4

urn:lsid:ipni.org:names:77209704-1

Figs 3A–D , 4

##### Type.

Japan. The Ryukyus: Miyako Islands, Miyako-jima Island, Gusukube, 5 April 2015, *Takuro Ito 2402* (holotype: TNS)

##### Diagnosis.

Sedum formosanum subsp. miyakojimense differs from its close relative S. formosanum subsp. formosanum in being perennial, polycarpic, and having lateral branches arising from the leaf axils.

##### Description.

Perennial herb, fleshy, glabrous. First year stem stout, erect, partly woody, 1–5 lateral branches in the leaf axils, 3–10 cm tall, with lax rosettes; rosettes 2.5–6 cm wide with 7–15 leaves. Flowering stems fleshy, 10–20 cm tall, base ca. 5 mm broad, yellowish green, erect or sprawling and creeping at base. Roots fibrous, sometimes adventitious at the leaf scar. Leaves alternate, occasionally verticillate, sessile, green or yellowish, flattish, ± thick, spatulate to oblanceolate, 1.1–3.1 cm long, 0.3–1.0 cm wide, apex rounded, base long, attenuate, margins entire. Inflorescences terminal, cymes, basically trifurcate with 3 primary axes, sometimes with 2, 4, or 5 primary axes; primary axis 2–8 cm long, ascending, 1 to several times irregularly and often unequally forking, with a flower at each fork, ultimate branches 1–2 cm long, 3–7 flowered; bracts leaf-like, smaller than cauline leaves. Flowers 5 (rarely 6)-merous, 7–11 mm wide, sessile. Sepals 5, free, yellowish green, fleshy, flattish, unequal in size, obovate to oblanceolate, 1.8–4.5 mm long, 1.2–3.3 mm wide, apex round or obtuse, base spurred. Petals 5, bright yellow, lanceolate, 4.6–6 mm long, 1.3–1.6 mm wide, apex acuminate, base slightly connate. Stamens 10, shorter than petals, 4.2–5 mm long, erect at flowering, two-whorled arrangement; anthers oblong-lanceolate, ca. 0.5 mm long, deep yellow before dehiscence. Pistils 5, 5.2–6.3 mm long; carpels 5, free, connate at the base, gibbous ventrally. Fruits star-shaped, follicle, erect, 5.3–6.8 mm long. Flowering in April to June.

##### Taxonomic note.

This new subspecies is classified in the sect. Sedum because of its adaxially gibbous carpels (Fu and Ohba 2001) (Fig. 3).

##### Etymology.

The epithet refers to the Japanese name of the type locality.

##### Distribution and habitat.

Endemic to the southeastern portion of Miyako-jima Island (The Ryukyus), on sunny, coastal limestone.

##### Additional specimens examined.

Japan. The Ryukyus: the Miyakojima Islands, Miyako-jima Island, Gusukube, 5 April 2015, *Takuro Ito 2403, 2408* (isotype: TNS).

##### Conservation.

**IUCN Red list category**: Critically Endangered (CR). The distribution of Sedum formosanum subsp. miyakojimense is restricted to only one location ca. 0.15 km^2^ in Miyako-jima Island, the Ryukyu Islands. The population of the species contains fewer than 200 mature individuals. The plant occurs on limestone rocks scattered in a private golf course, therefore, it is not formally protected. In the future, the population could become threatened, given ongoing land development for tourism in the Ryukyus. Because of the small population size (≤ 250 mature individuals) and small area of occupancy (≤ 10 km^2^), S. formosanum subsp. miyakojimense is classified as CR (IUCN 2019).

##### Japanese common name.

Miyako-hama-mannen-gusa (nov.).

### Possible biogeographical history of S. formosanum subsp. miyakojimense

The Ryukyu Islands, including Miyako-jima Island, experienced extensive land configuration changes throughout the Neogene and the Quaternary as a result of tectonic movements and sea level fluctuations induced by climatic oscillations (Kimura 2002; Osozawa et al. 2011; Furukawa and Fujitani 2014). Miyako-jima Island was likely originally located at the eastern margin of the continent, based on evidence of deposits derived from the continent during the late Miocene to Pliocene (Osozawa et al. 2011). The highest point on Miyako-jima Island is only 100 m above sea level; therefore, the entire island was likely submerged in the past under higher sea levels. Furthermore, the mud-dominant Shimajiri Group is mostly overlaid by the Ryukyu Group, which is composed of Pleistocene reef-complex deposits (Shokita et al. 2006). Although some endemic freshwater and terrestrial organisms, such as the Miyako toad (*Bufo gargarizans miyakonis* Okada) and the potamid crab (*Geothelphusa miyakoensis* Shokita, Naruse & Fujii) are reported from Miyako-jima Island (Shokita et al. 2006). Oshiro and Nohara (2000) suggested that the island likely reconnected to the Yaeyama Islands, located in the southern Ryukyus, during the last glacial period. However, these endemic species and their close relatives are not distributed in the Yaeyama Islands, and it is highly unlikely that they experienced long-range dispersal. Therefore, if these islands were connected during the last glacial period, it is unlikely that migration occurred from the Yaeyama Islands via a land bridge. Interestingly, the Shimajiri Group is partly exposed to the surface on the eastern portion of Miyako-jima Island (Shokita et al. 2006). This suggests that some areas of the island may have remained above water during sea level fluctuations, and freshwater species such as *G. miyakoensis*, freshwater red alga (*Thorea gaudichaudii* C. Agardh), and oriental weatherfish (*Misgurnus anguillicaudatus* Cantor) are only distributed in this area (Shokita et al. 2002, 2006). Collectively, this suggests that some organisms may have survived in isolation as relict populations, and further implies that the island may not have been entirely submerged in the past or, potentially, the existence of an ancient landmass adjacent to the island after its division from the continent (Shokita et al. 2006; Furukawa and Fujitani 2014). Previous molecular dating of East Asian *Sedum* species reported that *S. formosanum* diverged from the endemic Taiwanese species during the Pleistocene 1.41 Ma (0.79–2.25 Ma) (Ito et al. 2017b). Thus, it is reasonable to assume that S. formosanum subsp. miyakojimense may have diverged during the Pleistocene and has long since been genetically isolated from other species. Furthermore, S. formosanum subsp. miyakojimense is distributed in a restricted area on the eastern part of the island, in a similar location as the aforementioned endemic freshwater organisms. The discovery of a new endemic plant taxon, S. formosanum subsp. miyakojimense, on Miyako-jima Island is biogeographically important because it may imply that portions of the island remained above water over long time periods.

## Supplementary Material

XML Treatment for Sedum formosanum N.E. Brown., subsp. formosanum

XML Treatment for Sedum formosanum N.E. Brown., subsp. miyakojimense
